# 4R Tau Drives Endolysosomal Dysfunction in VCP‐Related Frontotemporal Dementia

**DOI:** 10.1002/alz70855_105883

**Published:** 2025-12-24

**Authors:** Christy Hung

**Affiliations:** ^1^ City University of Hong Kong, Hong Kong, Hong Kong

## Abstract

**Background:**

Frontotemporal dementia (FTD) and amyotrophic lateral sclerosis (ALS) share genetic and pathological features, with mutations in the *valosin‐containing protein (VCP)* gene implicated in both disorders. However, the mechanisms linking VCP mutations to neurodegeneration remain unclear. This study investigates how 4R tau dysregulation contributes to endolysosomal and autophagy dysfunction in human neurons.

**Method:**

Human induced pluripotent stem cell (hiPSC)‐derived cortical neurons carrying *VCP* mutations were analyzed for endolysosomal integrity, RNA‐binding protein (RBP) localization, and *MAPT* splicing. Antisense oligonucleotides (ASOs) were used to selectively increase 4R tau levels in control neurons to assess its impact on cellular homeostasis. Immunocytochemistry, western blotting, and proximity ligation assays were employed to evaluate tau phosphorylation, endolysosomal dysfunction, autophagic flux, and apoptosis.

**Result:**

*VCP* mutations caused nuclear dissociation of *fused in sarcoma (FUS)* and *splicing factor proline‐ and glutamine‐rich (SFPQ)*, leading to aberrant *MAPT* pre‐mRNA splicing and an increased 4R tau:3R tau ratio. These neurons exhibited enlarged endolysosomes, lysosomal membrane rupture, impaired autophagic degradation, endoplasmic reticulum stress, and apoptosis. Notably, ASO‐induced 4R tau expression in control neurons replicated these pathological phenotypes, confirming 4R tau as a driver of cellular dysfunction.

**Conclusion:**

Our findings demonstrate that 4R tau dysregulation contributes to neurodegeneration by disrupting endolysosomal and autophagic function in FTD. Targeting tau isoform balance may offer a promising therapeutic approach for *VCP*‐related FTD.

